# Day 7 blastocyst euploidy supports routine implementation for cycles using preimplantation genetic testing

**DOI:** 10.5935/1518-0557.20180089

**Published:** 2019

**Authors:** John B. Whitney, Katie Balloch, Robert E. Anderson, Nancy Nugent, Mitchel C. Schiewe

**Affiliations:** 1 Ovation Fertility, ART Lab, Newport Beach, CA 92663 USA; 2 Southern California Center for Reproductive Medicine, Newport Beach, CA 92663 USA

**Keywords:** embryo culture, Day 7, blastocyst, biopsy, PGT

## Abstract

**Objective:**

To determine if Day 7 blastocysts merit biopsy, vitrification and transfer consideration by contrasting their aneuploidy and implantation rates to Day 5 and 6 blastocysts.

**Methods:**

A total of 1,925 blastocysts were biopsied from 402 PGT-A cycles over a 12 to 16 month interval. All embryos were cultured under tri-gas, humidified conditions (37ºC) for up to 7 days (168 hours post-insemination). Biopsied blastocysts were vitrified and trophectoderm samples analyzed using NextGen sequencing. Single euploid embryo transfers were performed (n=254) using either a Day 5 (n=145), Day 6 (n=92) or a Day 7 blastocyst (n=16) post-warming. Euploidy rates and pregnancy outcomes were subsequently assessed and differences determined by day of development and blastocyst quality grade.

**Results:**

No differences were observed in implantation, pregnancy loss or ongoing pregnancy rates between Day 5 and Day 6 blastocysts. Development to Day 7 accounted for 6.6% of all blastocysts. Euploidy rates were higher in Day 5 blastocysts (53.5%; *p*<0.05) compared to Day 6 (40.4%) and Day 7 (35.9%). High implantation potential (56.3% to 79.3%) of vitrified-warmed euploid blastocyst occurred independent to the day of development. However, miscarriage/loss rates increased (22.2% *vs.* 2%; *p*<0.05) with Day 7 blastocysts, resulting in lower (*p*<0.05) live birth rates (43.8% *vs.* 67.4-77.2%).

**Conclusion:**

Culturing blastocysts to Day 7 has proven beneficial by achieving viable euploid embryos that would have otherwise been discarded. An extra Day of embryo growth allows select patients additional opportunities for *in vitro* development and possible healthy term live births.

## INTRODUCTION 

Human *in vitro* blastocyst development is highly dependent on variables inherent to the laboratories culture environment (Gardner, 2016; Swain *et al*., 2016). Environmental factors influencing embryo culture include, but are not limited to, temperature, pH, osmolality, growth promoters, protein supplementation, and the time allowed for development (Kovačič & Vlaisavljević, 2008; Christianson *et al*., 2014; Swain, 2015; Huang *et al*., 2016). Current culture interval parameters are broken down by hours post-insemination and typically conclude at 144 hours, or Day 6 (McArthur *et al*., 2005; Dahdouh *et al*., 2015). In lieu of the pioneering blastocyst culture efforts of Yves Menézo in the 1990's, investigators are again questioning if 144 hours of *in vitro* culture is an accurate measure of developmental potential when implementing routine blastocyst culture for all patients (Kovalevsky *et al.,* 2013; Capalbo *et al.,* 2015). Early human blastocyst culture efforts revealed that Day 7 blastocysts possessed reduced viability, but were capable of creating live births (Veiga *et al*., 1999; Richter *et al.,* 2006). Ultimately, a healthy baby is the end goal for any patient choosing to invest time, money and emotion into assisted reproductive technologies (Heijnen *et al*., 2004). Yet, for this goal to be realized a blastocyst must first be available.

The capability of the modern embryology lab has been revolutionized by the adoption of vitrification to reliably cryopreserve human blastocysts (Kuwayama *et al*., 2005; Loutradi *et al*., 2008; Schoolcraft & Katz-Jaffe, 2013; Schiewe *et al*., 2015; Gardner, 2016). Emphasis on the implementation of vitrification-all cycles to optimize uterine receptivity (Shapiro *et al.,* 2011; Desai *et al*., 2016), and in conjunction with preimplantation genetic testing for aneuploidy screening (PGT-A; Whitney *et al*., 2016) has placed pressure on the IVF laboratory to efficiently produce blastocysts (Roque *et al*., 2015; Desai *et al.,* 2016). Culture environment, specifically the type and conditioning of culture media, has been emphasized to maximize blastocyst yield per cycle and improve outcomes (Swain, 2015). Menezo's blastocyst culturing efforts with Day 7 embryos has seemingly been ignored by improvements in embryo culture medium. The acceptable timing for blastocyst development is portrayed as a fixed variable not usually evaluated beyond 144 hours (Barash *et al*., 2017).

An embryos ability to cleave and blastulate in an organized, predictable manner, as an indicator of developmental potential, does not guarantee normal chromosomal constitution or implantation. Current technologies implementing PGT-A have exhibited improvements in clinical pregnancies and live birth rates compared to untested first-time embryo transfers (Whitney *et al*., 2016). Some studies have also postulated that a quicker time to blastulation to be a predictor for implantation (Desai *et al.,* 2016), as well as slower blastocyst development correlating to higher aneuploidy (Kovalevsky *et al*., 2013). Given the complexity required to accurately choose a single embryo for transfer, we question whether todays' standard lab practices of 144 hr *in vitro* culture could be eliminating potentially viable embryos. Fundamentally, we questioned to what extent viable, euploid blastocysts can still be effectively produced by continuing culture an additional 24 hours to 168 hours post-insemination (Day 7).

## MATERIALS AND METHODS

### Population

Patients, averaging 37.3 years of age, electively chose IVF treatment with PGT-A of all viable embryos at a single IVF center/ART lab. All cycles performed standard controlled ovarian hyperstimulation protocols, established embryo culture practices, and intracytoplasmic sperm injection (ICSI). Cycles initiated January 1^st^, 2015 to March 1^st^, 2016 were enrolled in an observational study to assess Day 7 blastocyst aneuploidy and implantation potential. Patients were made aware of embryo genetic outcomes and the transfer selection was made by patient preference or available top quality euploid embryo. No randomization or control treatments were performed as this was a retrospective observational analysis of standard IVF applications following informed patient consent and prior Investigational Review Board approval.

### Embryo Culture and Blastocyst Grading

Using MCO-5M mini Sanyo/Panasonic tri-gas incubators (5% 0*2*/5.3-6.0% CO*2*), we group cultured up to 5 embryos per 25µL droplet of Global™ medium (LG; Life Global, Guilford, CT) supplemented with 7.5% synthetic protein supplement under Ovoil™ (Vitrolife, Englewood, CO) until blastocyst biopsy (Whitney *et al*., 2016). All oocytes retrieved were evaluated for maturity and had ICSI performed 2-6 hours post-egg retrieval. Embryos were initially evaluated at 14-18 hours post-insemination for fertilization. All embryos exhibiting normal 2PN fertilization and all failed fertilization (0PN) zygotes were cultured an additional 48 hours. On Day 3, 72 hours post-egg retrieval, embryos were evaluated for cleavage development and quality. All cleaved embryos having 3 cells or more were laser zona dissected using a 1410-nm diode laser (Zilos-tk™; Hamilton Thorne, Beverly, MA) and embryo incubation continued an additional 48 hours to Day 5 (Whitney *et al*., 2015). At 120 hours post-egg retrieval, or Day 5, all embryos were assessed for blastocyst development and quality graded (QG) using a modified Gardner system (Whitney *et al*., 2015). In short, pre-maturely herniating blastocysts were classified as QG3= <10% trophectoderm hatching, QG4=10-50% hatching and QG5=>50% hatching. Full blastocysts (QG3), expanded blastocysts (QG4), hatching blastocysts (QG5) and hatched blastocysts (QG6) were biopsied and vitrified. Inner cell mass (ICM) and trophectoderm were independently graded from top quality "A" (excellent) to good-fair quality "B" and fair-poor quality "C" with the first letter in the grade assigned to the ICM and the second to trophectoderm (Gardner & Schoolcraft, 1999). In brief, "A" ICM possess numerous, tightly packed cells, while "A" trophectoderm have a highly cellular, cohesive layer free of irregularities. "B" and "C" quality ICM and trophectoderm possess fewer, more loosely conjoined cells, as well as varying levels of notable defects and unincorporated cells. Typically, a grade of 3BB or better was required to initiate biopsy, however lower quality blastocysts possessing a "C" grade may have been incorporated into patient sub-groups, especially in patients possessing few blastocysts. Embryos not achieving the minimum full blastocyst stage on Day 5 were cultured an additional 24 to 48 hours. Embryos were only assessed once between 6am-12pm. At 144 hours, or Day 6, the same evaluation was performed and blastocysts biopsied. If any of the remaining cohort of embryos had at minimum a morula, all embryos continued an additional 24 hours of *in vitro* culture. A final assessment was made 168 hours post-ICSI, or Day 7, which was equilvalent to 206 to 210 hours post-hCG administration. All embryos not achieving a full blastocyst stage were discarded. All biopsied embryos were vitrified pending PGT-A results.

### Blastocyst Biopsy and PGT-A

The zona opening created on Day 3 allowed trophectoderm to prematurely rupture through a 10-12µm furrow in the zona. The Zilos-tk™ laser was used for biopsying, as reported previously (Whitney *et al*., 2016). In short, a combination of laser pulses and mechanical separation was applied to isolate 3-10 trophectoderm cells. All biopsy samples were aseptically pipette into individual PCR tubes, frozen, shipped on dry ice and analyzed for PGT-A using Next Generation Sequencing at either Genesis Genetics (Plymouth, MI) or Ovation Fertility Genetics (Henderson, NV). Both facilities used the VeriSeq platform (Illumina, San Diego, CA). Confirmed euploidy results were required to proceed with a vitrified-warmed embryo transfer. All aneuploid embryos were discarded and removed from frozen inventory per patient consent. Mosaic profiles were reported as aneuploid according to standard lab calling policies and no mosaic outcome embryos were electively transferred.

### Day 7 blastocyst euploidy supports

Blastocysts were vitrified using microSecure-VTF in glycerol/EG, non-DMSO VTF solutions (Innovative Cryo Enterprises, Linden, NJ; Schiewe *et al*., 2015; 2017). Aseptic microSecure VTF was performed using: a 3-step dilution (5 min/5min/1min); individual blastocysts were loaded into 300 µm ID flexipettes (Cook Medical, Spencer, IL; 3 µl volume); flexipettes were then dried and inserted tip first into prelabeled 0.3ml CBS™ embryo straws; the straw weld sealed; and plunged directly into LN_2_ (Schiewe *et al*., 2015). Rapid warming was achieved by direct placement of the vitrified flexipettes into a 37ºC 0.5M sucrose bath (see video: Schiewe *et al*., 2017). Within 10 sec, each blastocyst was pipette directly from the flexipette into an open 200 µl droplet of 1.0M sucrose solution and then transferred into 100 µl droplets under oil for 3min intervals. Embryos were serially diluted in declining sucrose solutions (T1-T4; 3 min/step at 21ºC), before isotonic equilibration in Hepes-LG medium (5 min at 37ºC). Warmed blastocysts were then cultured in LG medium + protein for 1-3 hr prior to vitrified ET (VFET). As long as blastocysts were osmotically reactive to post-warming dilutions and appeared viable, re-expansion was not a requirement for ET to proceed.

All VFET cycles involved hormone replacement cycles using oral estradiol, estradiol patches or intramuscular (i.m.) estradiol valerate followed by i.m. progesterone in oil. Progesterone in oil was started when endometrial thickness was > 8mm after documentation of serum progesterone level of <1.0 ng/ml. VFET was performed after 5.5 Days of intramuscular progesterone administration. All transvaginal ultrasound guided ET procedures were performed by a single physician (Anderson *et al*., 2002). Pregnancies were initially tested 10d post-ET and implantation subsequently assessed by transvaginal ultrasound beginning 4 weeks later. Live births were confirmed by written or oral communication with patients.

## Statistical Analysis

Blastocyst development was calculated by the successful biopsy of a blastocyst on Day 5, 6 or 7 per 2PN achieved from a single patient retrieval cycle (Table 1). Aneuploidy percentages were determined by the total euploid reported minus the total tested for each day of culture. Initial comparisons for implantation, clinical pregnancies, live births and spontaneous abortions were calculated per transfer attempt (Table 2). Additionally, data were further sub-divided by blastocyst quality grades (Table 3). Chi-squared analyses were performed to contrast differences (*p*<0.05) in blastocyst development, aneuploidy, implantation and live birth outcomes.

**Table 1:** Blastocyst development and euploidy status by day of *in vitro* culture.

	Day 5	Day 6	Day 7
# Biopsied	879	918	128
% Blastocyst Yield	45.7%	47.7%	6.6%
# Normal	470	371	46
% Euploid	53.5%^a^	40.4%^b^	35.9%^b^

a,b Row values with different superscripts are different (*p*<0.05)

**Table 2:** Pregnancy outcomes of euploid blastocysts biopsied and vitrified on either Day 5, 6 or 7 of *in vitro* culture.

	Day 5	Day 6	Day 7
# of Embryos Transferred	145	92	16
# of Implantations	115	63	9
Implantation Rate	79.3%	68.5%	56.3%
Spontaneous Abortion Rate	2.6% (3/115)^a^	1.6% (1/63)^a^	22.2% (2/9)^b^
Live birth rates	77.2% (112/145)^a^	67.4% (62/92)^a^	43.8% (7/16)^b^

a,b Combined row values with different superscripts, within sub-sections, were different (p<0.05).

## RESULTS

A total of 1,925 blastocyst embryos were biopsied between the months of January and December of 2015. Blastocyst biopsy was performed on Day 5, 6 and 7 of embryonic development. With a preference toward antagonist stimulated cycles, a mean of 13.1 mature oocytes (MII) per-cycle was produced with a normal fertilization rate of 75% and an average yield of 9.9 zygotes per-cycle for culture to the blastocyst stage. Originating from 315 cycles, 887 (46%) of the blastocysts were euploid after PGT-A determination (Table 1). Ninety-two percent of the cycles created a blastocyst for PGT-A determination, with an average of 6.6 blastocysts tested per biopsy patient. A normal embryo was produced in 77% of the IVF cycles, with an average of 2.3 euploid blastocysts per-cycle tested. A total of 253 single euploid embryo transfers (SEETs) were performed resulting in 187 implantations (74%) and 181 singleton live births (72%; Table 2). The euploidy of embryos blastulating on Day 5 was significantly higher than embryos biopsied on Day 6 and Day 7 (*p*<0.05), however, there was no significant difference in implantation rates amongst embryos blastulating on Day 5 (79.3%), Day 6 (68.5%) or Day 7 (56.3%; Table 2).

Pregnancy loss or ongoing pregnancy rates between Day 5 and Day 6 blastocysts was not different (*p*>0.05), however Day 7 blastocysts experienced significant fetal losses (22.2%) and lower (*p*<0.05) live birth rates (Table 2). Late blastocyst formation on Day 7 only accounted for 6.5% of all development, yet interestingly still yielded euploid blastocysts capable of implantation and normal development. Day 5 blastocysts had an overall higher euploidy rate than Day 6 and Day 7 which exhibited no difference (Table 1). Morphological grade ranking, was predictive of aneuploidy in that high quality Day 5 blastocysts with Grade "A" trophectoderm achieved statistically higher euploidy rates compared to Day 6 (371/ 634, 58.5% and 234/496, 47.2%, respectively), while fair to poor quality embryos had increased aneuploidy independent of day of development (Table 3). Day of development was correlated to embryo grade, with Day 5 producing 90.4% excellent-to-good quality blastocysts (AA, BA, AB), compared to 70% and 45.2% for Days 6 and 7, respectively. Yet, the implantation potential of an embryo was unaffected by day of development when transferring euploid embryos (Table 2).

**Table 3:** Euploidy status of blastocysts based on embryo quality grade and day of development.

Grade	Day 5	Day 6	Day 7*
# Grade AA	556	330	19
# Euploid	325	165	12
% Euploid	58.5% ^a^	50.0% ^b^	63.2%
# Grade AB	161	146	16
# Euploid	62	54	7
% Euploid	38.5%	37.0%	43.8%
# Grade BA	78	166	22
# Euploid	46	69	9
% Euploid	59.0% ^a^	41.6% ^b^	40.9%
# Grade BB	77	220	41
# Euploid	33	62	15
% Euploid	42.9% ^a^	28.2% ^b^	36.6%
#Grade BC	2	17	5
# Euploid	0	5	0
% Euploid	0.0%	29.4%	0.0%
# Grade CB	3	24	19
# Euploid	2	8	2
% Euploid	66.7%	33.3%	10.5%
# Grade CC	0	3	1
# Euploid	0	2	0
% Euploid	0.0%	66.7%	0.0%
# Grade CA	1	10	5
# Euploid	1	5	1
% Euploid	100.0%	50.0%	20.0%
# Grade AC	1	2	0
# Euploid	1	1	0
% Euploid	0.0%	50.0%	0.0%

a,b Row values with different superscripts are different (*p*<0.05)

* Day 7 data were not statistically analyzed due to low sample sizes.

In reviewing the delayed development of Day 7 blastocysts, 68% were ≤morulae stage (8% pre-compaction), 28% were partial/complete early blastocysts and 4% were ≤2BB stage embryos on Day 5. On Day 6, 12% were still ≤morulae stage, while 48% were partial/complete early blastocysts, 35% were 2AA-CC quality and 5% more developed blastocysts on Day 6, but their quality grade was <BB. About 8% of the embryos exhibited no change in development between Day 5 to Day 6. Thus, inclusion criteria for Day 7 biopsy was a progression or improvement in blastocyst development or quality grade, respectively. On Day 7, 42% of the embryos were fully hatched, 38% classified as hatching (QG=5) and 23% exhibited progressive herniation of the trophectoderm as grade 3 and 4 blastocysts.

## DISCUSSION

Modern embryology methods have placed an emphasis on optimizing *in vitro* embryo culture conditions to produce more fast growing, higher quality, physiologically normal blastocysts (Gardner, 1998; Wale & Gardner, 2016). Current societal and industry pressures for single embryo transfers have increased the need for accurate selection criteria to best optimize pregnancy outcomes (Yang *et al.*, 2012). Our study identifies that *in vitro* blastocyst development may initiate between 96 and 172 hours post-insemination with emphasis on Day 5, or 120 hours post-insemination, top morphologically graded blastocysts (Barash *et al.,* 2017). This long held philosophy was supported by our data, but not definitively so. Our study indicates Day 5 blastocysts with "A" quality trophectoderm produce higher euploidy rates, further supporting previous assertions that an "A" trophectoderm grade is more predictive of pregnancy success than ICM grades (Ahlström *et al*., 2013; Whitney *et al*., 2015). Although euploidy on Day 5 is increased, this study indicates that ploidy status is the definitive value for implantation success, not the day of development.

Our study supports the observations of Capalbo *et al*. (2014, 2015) relative to the importance of morphology and developmental progress, with the implantation potential of euploid blastocysts being independent of morphology and *in vitro* culture interval. Both groups observed no difference in implantation for euploid embryos generated on Day 5, 6 or 7. The differences observed in Day 7 euploidy rates between Capalbo *et al*. (2014) and our investigation (43.5% *vs.* 35.9%, respectively), may simply reflect our willingness to have biopsied more poor quality embryos. In either case, we achieved similar ongoing implantation/live birth outcomes using euploid Day 7 blastocysts (50% to 43.8%), which was significantly better than that of untested Day 7 blastocysts (27%; Kovalevsky *et al*., 2013). In contrast to the valuable multi-center/multi-physician observational studies of Capalbo *et al*. (2014, 2015), the higher overall implantation success observed in our study on Days 5 and 6 may reflect a benefit to reduced confounding procedural variables associated with a single physician IVF center. In the laboratory, differences in our VTF procedure (i.e., use of a non-DMSO solution; Schiewe *et al*., 2015) or perhaps biopsy technique (i.e., Day 3 pre-laser zona dissection; Whitney *et al*., 2016) may have benefitted embryo well-being. In particular, we hypothesize that recently biopsied trophectodermal cells may be susceptible to DMSO exposure adversely affecting their normal functionality, without a pre-VTF equilibration period (>2 to 3 hours). We believe that glycerol/EG may be a safer and more effective VTF solution for blastocysts. In this study, SEET procedures were performed without regard to whether blastocyst re-expansion post-warming occurred. Further investigations focused on post-biopsy pre-VTF equilibration intervals and post-warming blastocyst re-expansion time could add to our understanding of the developmental competence and implantation potential of PGT-A tested blastocysts.

Although human blastocysts can sustain *in vitro* development up to Day 14 (Edwards *et al*., 1981; Shahbazi *et al*., 2016), they typically initiate implantation (i.e., attachment) by Day 8 (Shahbazi *et al*., 2016). Therefore, it is reasonable to assume that Day 7 is the last day our current static culture environment should sustain pre-transfer embryo growth. Not surprisingly, Day 7 blastocyst yield is significantly reduced in comparison to Day 5 and 6, yet Day 7 euploid blastocysts have proven viable and implanted. Considering human trophectoderm cells are programmed to invade the endometrium upon hatching, theoretically, the more robust the trophectoderm the greater its chance to initiate pregnancy (Ahlström *et al*., 2013). Day 7 blastocysts can potentially promote implantation but are vulnerable to increased pregnancy loss, perhaps due to a lack of cellular vigor to offset their delayed growth or trophectoderm degeneration due to the extended length of culture. Alternatively, our low patient numbers may not have accurately reflected the situation, or perhaps uterine asynchrony may have contributed to the problem warranting a possible change in the luteal support protocol when transferring a Day 7 blastocyst.

Ten patients achieved a transferable euploid embryo that would have been otherwise discarded if we did not grow their embryo(s) to Day 7. Over a 14-month interval, this study yielded 3 healthy term births, without complications or known abnormalities, which would not have been available under previous treatment protocols with culture ending at 144 hours. Our results support Yves Menezo's original assertion of the potential merits obtained applying Day 7 blastocyst culture. Furthermore, as shown by Capalbo *et al.* (2014, 2015), the routine implementation of Day 7 culture is particularly important for IVF cycles experiencing delayed blastocyst development, such as vitrified-warmed oocyte cycles, or any cycle seeking aneuploidy determination following substandard Day 6 development. We acknowledge the euploidy rate and embryo yield from Day 7 is low and requires additional staff time and effort. Nonetheless, our goal in the IVF lab is to afford our patient's every opportunity for a live birth, and Day 7 culture has proven beneficial to achieve this objective.
